# The transition from cannabis induced psychosis to primary psychosis: factors that question the validity of the diagnosis “cannabis-induced psychosis”

**DOI:** 10.1192/j.eurpsy.2025.48

**Published:** 2025-08-26

**Authors:** E. Rognli

**Affiliations:** Department of Mental Health and Addiction, Oslo University Hospital, Oslo, Norway

## Abstract

**Abstract:**

Background: Substance-induced psychosis is an acute psychotic condition occurring after substance use, where the psychotic symptoms are alleviated with abstinence. Some of those diagnosed with substance-induced psychosis later develop schizophrenia, and this transition occurs most often for psychosis induced by cannabis. This talk will present results from a register-based study investigating transition rates. The results will be discussed together with other factors that together question the validity of the diagnostic entity of “cannabis-induced psychosis” Method: In our study, we used data from National Patient Register in Norway from 2010 to 2015 to estimate the cumulative hazard for transition from any substance-induced psychosis (F1x.5) and cannabis-induced psychosis (F12.5) to schizophrenia spectrum disorder (F20, F22 and F23). Results: The six-year cumulative hazard for transition from substance-induced psychosis to schizophrenia was 27.6% (25.6-29.7) for any SIP, and highest for those with cannabis-induced psychosis, 36% (95% CI 31.4-41.0). Conclusion: Cannabis-induced psychosis constitutes a significant risk for later schizophrenia. This raises a question of whether the initial diagnosis of CIP was correct, or whether these may have been cases of a development of primary psychosis concealed by cannabis use. Further, the term “cannabis-induced psychosis” places a major explanatory emphasis on the substance use, ignoring other potential contributing factors such as vulnerability including genetic disposition for psychosis.

**Disclosure of Interest:**

None Declared

